# Biogenic Synthesis, Structural Characterization, and Biological Evaluation of Nanoparticles Derived from *Chlorella vulgaris* Ethanolic Extract

**DOI:** 10.3390/nano16030177

**Published:** 2026-01-28

**Authors:** Alexandra Ivanova, Mina Todorova, Dimitar Petrov, Vera Gledacheva, Iliyana Stefanova, Miglena Milusheva, Valeri Slavchev, Gabriela Kostadinova, Zhana Petkova, Olga Teneva, Ginka Antova, Velichka Yanakieva, Slava Tsoneva, Daniela Karashanova, Krastena Nikolova, Stoyanka Nikolova

**Affiliations:** 1 Department of Organic Chemistry, Faculty of Chemistry, University of Plovdiv, 4000 Plovdiv, Bulgaria; ivanova.aleksandra@uni-plovdiv.bg (A.I.); minatodorova@uni-plovdiv.bg (M.T.); gabrielitta03@gmail.com (G.K.); 2 Department of Physical Chemistry, Faculty of Chemistry, University of Plovdiv, 4000 Plovdiv, Bulgaria; petrov_d@uni-plovdiv.bg; 3 Department of Medical Physics and Biophysics, Faculty of Pharmacy, Medical University of Plovdiv, 4002 Plovdiv, Bulgaria; iliyana.stefanova@mu-plovdiv.bg (I.S.); valeri.slavchev@mu-plovdiv.bg (V.S.); 4 Department of Bioorganic Chemistry, Faculty of Pharmacy, Medical University of Plovdiv, 4002 Plovdiv, Bulgaria; miglena.milusheva@mu-plovdiv.bg; 5 Department of Chemical Technology, Faculty of Chemistry, University of Plovdiv, 4000 Plovdiv, Bulgaria; zhanapetkova@uni-plovdiv.bg (Z.P.); olga@uni-plovdiv.bg (O.T.); ginant@uni-plovdiv.bg (G.A.); 6 Department of Microbiology, Technological Faculty, University of Food Technologies, 4002 Plovdiv, Bulgaria; yanakieva_vili@abv.bg; 7 Department of Analytical Chemistry and Computer Chemistry, University of Plovdiv, 4000 Plovdiv, Bulgaria; slava.tsoneva@uni-plovdiv.bg; 8 Institute of Optical Materials and Technologies “Acad. J. Malinowski”, Bulgarian Academy of Sciences, 1113 Sofia, Bulgaria; adi@iomt.bas.bg; 9 Department of Physics and Biophysics, Faculty of Pharmacy, Medical University of Varna, 84 Tzar Osvoboditel, 9000 Varna, Bulgaria; krastena.nikolova@mu-varna.bg

**Keywords:** *Chlorella vulgaris*, silver nanoparticles, tocopherols, carotenoids, chlorophylls, antimicrobial, antioxidant activity

## Abstract

*Chlorella vulgaris* is a microalga with well-established nutritional, antioxidant, anti-inflammatory, and antibacterial potential. The current study aimed to explore the green synthesis of silver nanoparticles (AgNPs) using the ethanolic extract of *C. vulgaris* and to assess how nanoparticle formation affects the chemical composition, antimicrobial potential, antioxidant capacity, and spasmolytic activity of the extract, building on earlier evidence for its modulatory effects on gastrointestinal smooth muscle. Even though AgNPs from *Chlorella* have been obtained previously, to the best of our knowledge, their spasmolytic activity has not been evaluated. To assess their properties and stability, ATR-FTIR, TEM images, XRD, DLS, and zeta potential were used. The obtained AgNPs were mostly spherical (with a diameter between 10 and 50 nm) and showed good colloidal stability. The synthesis of AgNPs resulted in significant changes in lipid composition, pigment content, and fatty acid profiles, including a decrease in total chlorophylls and an increase in mono- and polyunsaturated fatty acids. The biosynthesized AgNPs showed moderate to strong antibacterial activity against a variety of Gram-positive and Gram-negative bacteria, yeasts, and fungi. The most pronounced inhibitory effect was observed against *A. niger* and *P. chrysogenum*. In ex vivo organ bath experiments, AgNPs modulated the contractile activity and the spasmolytic profile of isolated rat gastric smooth muscle compared with *C. vulgaris* extract. These results demonstrate that green-synthesized AgNPs present systems with altered smooth muscle activity and improved antibacterial qualities, underscoring their potential for use in functional foods, nutraceuticals, and gastrointestinal therapeutics.

## 1. Introduction

*Chlorella* spp. are among the many types of microalgae that demonstrate promise as sources of commercially significant compounds [1]. They are also well-known for their many metabolites, which are used in the food, feed, pharmaceutical, and energy industries [2]. For example, their nutritional benefits provide additional advantageous properties such as antiviral, antibacterial, antifungal, anticancer, anti-inflammatory, and antioxidant properties [3].

This genus is capable of both autotrophic and heterotrophic growth. According to Hu et al. [4], this microalga can grow in the dark using simple organic matter as a source of energy. *Chlorella* is also known for its ability to eliminate organic pollutants from water bodies or wastewater that have been contaminated by human and animal hygiene products, such as disinfectants, insect repellents, UV filters, shampoos, lipsticks, flavorings, moisturizers, detergents, soaps, toothpaste, cloth cleaners, and more [2].

One of the microalgae species that has been most extensively studied is *C. vulgaris* due to its commercial viability and resistance to infections and adverse environmental conditions [5]. *Chlorella vulgaris* belongs to the Plantae Kingdom, *Chlorophyta* division, *Trebouxiophyceae* class, *Chlorococcales* order, *Chlorellaceae* family, of the genus *Chlorella*. The microalgae are found in marine and freshwater environments, on surfaces, in sediments, and throughout the water column [6]. It has a green alga that is spherical, unicellular, eukaryotic, and capable of photosynthesis. They can range in size from one to ten microns [7]. The color of the microalgae can be seen to change to a dark green when the cells are damaged or in a state of death [7]. The cell wall, which has a maximum diameter of 21 nm, is known for its resistance to heavy metals and for providing chemical and mechanical protection [8].

It has a number of significant benefits; it can grow under regulated circumstances without any contamination, meaning its growth is independent of weather, climate, or light sources. These necessitate thorough cultivation and ongoing sterility monitoring. *C. vulgaris* can develop in colonies of up to 64 cells or in isolation [5].

In our previous research, we examined the anti-inflammatory and spasmolytic activity of commercial *C. vulgaris* extracts to assess their potential as functional food ingredients for digestive health [9]. A logical extension of this research is the green synthesis of AgNPs using *C. vulgaris* extracts, aiming to compare their spasmolytic activity with the original extracts, as well as to establish the impact on the extracts’ antispasmodic activity.

Silver has been valued for its antimicrobial activity for centuries. It has been used to store water and to heal burns, wounds, ulcers, and infant eye infections [10]. Silver’s active surface is increased, and its ability to penetrate bacteria is improved when it is reduced to a nanometer size (1–100 nm). AgNPs work by attaching themselves to the bacterial cell wall, increasing the release of reactive oxygen species, rupturing membranes, interacting with respiratory enzymes and phosphate groups of proteins and DNA, disrupting cellular components, destroying cells, allowing cell contents to leak out, or controlling important gene expression [10,11,12].

Physical, chemical, and biological techniques can be used to create AgNPs. Each of these techniques creates nanoparticles by reducing silver ions from salt and then allowing atoms to self-assemble [13]. Green synthesis techniques are eco-friendly, employ renewable energy sources, and use less harmful chemicals. In green synthesis, organisms like plants, algae, bacteria, and fungi reduce silver ions [14]. Living organism extracts produce nanoparticles with improved antibacterial, antifungal, drug transport, and photodegradation capabilities [15,16,17]. Amino acids, polyphenols, flavonoids, polysaccharides, terpenoids, alkaloids, and other naturally occurring reducing and stabilizing agents of natural origin are used in this environmentally friendly method [14]. Recent studies have highlighted the growing biomedical relevance of green-synthesized metal nanoparticles, particularly due to their enhanced biocompatibility and multifunctional biological effects. Singaravelu et al. demonstrated that green-synthesized metal nanoparticles exhibit significant antimicrobial and tissue-regenerative properties, emphasizing their promise for biomedical applications such as wound healing and infection control [18]. In parallel, Balaji et al. emphasized that plant-mediated synthesis of metal and metal oxide nanoparticles represents a sustainable and effective strategy to counter antimicrobial resistance, while simultaneously reducing environmental burden compared to conventional synthetic approaches [19].

The microalga *Chlorella vulgaris* contains a variety of bioactive compounds that are used in the AgNP synthesis, including polysaccharides (starch and cellulose), vitamins, proteins, lipids, antioxidants (polyphenols and tocopherols), and pigments like carotenoids (carotene and xanthophyll), chlorophylls and phycobilins, phycocyanin, and phycoerythrin [20,21]. Algal extracts have a favorable nutritional profile due to their high quantity of powerfully stabilizing polysaccharides, lack of potentially harmful chemicals, and inclusion of a variety of substances such as peptides and unsaturated fatty acids [22]. Microalga-derived biomolecules, such as pigments, proteins, and polysaccharides, play a critical role in nanoparticle stabilization and biological activity, as demonstrated for both *Chlorella vulgaris* and *Spirulina platensis* [23]. Through surface modifications employing different functional groups and biomolecules, nanoparticles are enhanced with distinctive features by using algae biomass in the manufacturing process.

Phytochemicals are proven to possess a range of valuable pharmacological characteristics, such as antibacterial, anti-inflammatory, and antioxidant effects. These properties allow plant extracts to play catalytic and stabilizing roles in the creation of nanoparticles [24]. For example, plant extracts high in polyphenols have the ability to modulate the rate of reaction during the synthesis of AgNPs and are crucial in determining the size of the final nanoparticles. Additionally, flavonoids can adsorb onto nanoparticle surfaces, changing their characteristics for a range of uses [25]. A recent bibliometric analysis by Aliero et al. further confirms the rapidly increasing scientific interest in green-synthesized silver nanoparticles, particularly in relation to their antibacterial activity. The analysis highlights a steady growth in publications over the past decade, underscoring both the relevance and translational potential of biogenic AgNPs in addressing current biomedical and environmental challenges [26].

Previously, the green synthesis of AgNPs using green alga (*Chlorella vulgaris*) was reported, while AgNPs were used as a catalyst for the synthesis of quinoline derivatives [27]. They were also synthesized for application in photocatalytic dye degradation [28]. Other studies have reported the response of algae to metal nanoparticles under the modulation of nitric oxide [29]. To the best of our knowledge, the antispasmodic properties of AgNPs made from extracts of *Chlorella vulgaris* have not yet been studied.

Closely related studies have demonstrated that microalgae-derived biomolecules play a decisive role in silver nanoparticle nucleation, growth, and stabilization. In particular, *Spirulina platensis* and *Chlorella vulgaris* share comparable pigment, protein, and polysaccharide profiles, which critically influence nanoparticle size distribution, surface chemistry, and biological functionality. A recent study by Sidorowicz et al. systematically evaluated AgNPs synthesized using *Spirulina platensis*-derived biomolecules, reporting that chlorophylls, phycobiliproteins, and polysaccharides act synergistically as reducing and capping agents, resulting in stable nanoparticles with pronounced antimicrobial activity [23].

Compared to *Spirulina*, *Chlorella vulgaris* is characterized by a higher relative abundance of chlorophylls, lipophilic antioxidants, and neutral polysaccharides, which may contribute to distinct nanoparticle–biomolecule interactions and, consequently, to differences in physicochemical properties and biological responses. Therefore, exploring AgNPs derived specifically from *Chlorella vulgaris* provides valuable insight into how subtle variations in microalgal biochemical composition translate into functional nanoparticle systems.

Addressing this knowledge gap, the present study aimed to synthesize silver nanoparticles via a green approach using *Chlorella vulgaris* ethanolic extract and to evaluate how nanoparticle formation influences their spasmolytic and related pharmacological effects.

## 2. Materials and Methods

### 2.1. Plant Material and Chemicals

The *Chlorella* sample was bought from the local market in Bulgaria (Dragon Supefoods, Plovdiv, Bulgaria) [9].

All chemicals, including silver nitrate (AgNO_3_), acetylcholine chloride, ethanol, and analytical reagents, were of analytical grade and purchased from Sigma-Aldrich (Merck, Darmstadt, Germany), unless otherwise stated.

### 2.2. Extract Preparation and AgNPs from Chlorella vulgaris (Chlorella-AgNP) Synthetic Procedure

An amount of 1 g of powdered *Chlorella vulgaris* was subsequently submerged in 10 mL of 95% ethanol in a solid-to-solvent ratio of 1:10 (*w*/*w*). The ultrasound-assisted extraction of biologically active substances from *Chlorella* was carried out in an ultrasonic bathat 40 °C for 40 min. The extraction procedure was repeated twice, and the ethanol extracts were filtered through filter paper. The resultant leaf infusion was filtered using Whatman paper. An amount of 1 mL of extract was mixed with 9 mL of a 10 mM AgNO_3_ solution. The reaction mixture was incubated for 4 min at 40 °C under continuous stirring. The formation of AgNPs was monitored visually by a gradual color change from pale yellow to dark brown.

### 2.3. Characterization of the AgNP Analytical Techniques

After obtaining the *Chlorella*-AgNPs, they were analyzed through FTIR-ATR, transmission electron microscopy (TEM), XRD, dynamic light scattering (DLS), and zeta potential determination.

#### 2.3.1. FTIR Spectra

ATR spectra were determined on a VERTEX 70 FT-IR spectrometer (Bruker Optics, Ettlingen, Germany). The spectra were collected from 600 cm^−1^ to 4000 cm^−1^ with a resolution of 4 nm and 32 scans. The instrument was equipped with a diamond-attenuated total reflection (ATR) accessory (PIKE MIRacle™ Single Reflection ATR device, ZnSe crystal, Madison, WI, USA). The spectra were analyzed with the OPUS-Spectroscopy Software, Bruker (Version 7.0, Bruker, Ettlingen, Germany).

#### 2.3.2. TEM

The TEM micrographs were registered by means of the Orius SC200D Model 833 CCD camera (Gatan Inc., Pleasanton, CA 94588, USA) of the JEOL JEM 2100 HRTEM microscope (JEOL Ltd., Tokyo, Japan) at an accelerating voltage of 200 kV. In the preliminary preparation of TEM samples, the initial nanoparticle suspension was dropped onto a standard copper grid covered by an amorphous carbon film and then dried under ambient conditions.

#### 2.3.3. DLS and Zeta Potential

Dynamic light scattering (DLS) and zeta potential measurements were performed using a Brookhaven BI-200 goniometer equipped with a vertically polarized He–Ne laser (λ = 632.8 nm, 35 mW) and a Brookhaven BI-9000 AT digital autocorrelator (Brookhaven Instruments, Holtsville, NY, USA). Measurements were conducted on dilute aqueous dispersions in the concentration range of 0.056–0.963 mg mL^−1^. DLS analyses were performed at scattering angles between 50° and 130° and at temperatures of 25, 37, and 65 °C. All reported particle sizes correspond to the hydrodynamic diameter obtained from intensity-based size distributions, and polydispersity index (PDI) values were automatically calculated by the instrument software. Zeta potential measurements were carried out at 25 °C in triplicate. The system allows measurements of ζ potential in the range of −200 mV to +200 mV.

#### 2.3.4. X-Ray Diffraction (XRD)

The degree of crystallinity of the synthesized nanoparticles was studied by X-ray powder diffractometry. The diffraction patterns of AgNPs were recorded at a 2θ range from 10° to 80° using a SIEMENS D500 X-ray powder diffractometer (KS Analytical Systems, Aubrey, TX, USA). All the measurements were performed at a voltage of 35 kV and a current of 25 mA. The monochromatic X-rays (1.5406 Å) were generated by a Cu-anticathode (K_α1_).

### 2.4. Chemical Composition

#### 2.4.1. Glyceride Oil Content

The glyceride oil was obtained through hexane extraction of the ground material using the Soxhlet apparatus. The extraction duration was 8 h at 70 °C. After that, the solvent was evaporated, and the glyceride oil content was determined gravimetrically [30].

#### 2.4.2. Fatty Acid Composition

Fatty acid composition of glyceride oils was determined through gas chromatography (GC). Briefly, the oil was subjected to transesterification with methanol in the presence of sulfuric acid [31,32]. The analysis was performed on an Agilent 8860 system (Santa Clara, CA, USA) with a flame ionization detector (FID) and a capillary column DB-Fast FAME (Agilent, Santa Clara, CA, USA) (30 m × 0.25 mm × 0.25 μm). The oven temperature was set at 70 °C for 1 min and then increased to 250 °C at a rate of 5 °C per minute; finally, this temperature was held for 3 min. The injector’s and detector’s temperatures were 270 °C and 300 °C, respectively. For identification of the components, a standard FAME mixture containing 37 compounds (Supelco, Bellefonte, PA, USA) was analyzed under the same conditions.

#### 2.4.3. Total Pigment Content

Total chlorophyll content was determined spectrophotometrically at a wavelength of 670 nm according to the method described by Borello and Domenici [33].

For the analysis of chlorophyll a (C_a_), chlorophyll b (C_b_), and carotenoids, the absorbance (A) of the 95% ethanol extract was measured at three wavelengths (470 nm, 664 nm, and 648 nm). The pigment content was calculated according to Equations (1)–(4) [34].

(1) $$C_{\mathrm{clorophyla}}\;(µg/\mathrm{mL})=13.36A_{664}\;-\;5.19A_{648}$$

(2) $$C_{\mathrm{clorophylb}}\;(µg/\mathrm{mL})=27.43A_{645}\;-\;8.12A_{664}$$

(3) $$C_{\mathrm{clorophyla}+b}\;(µg/\mathrm{mL})=5.24A_{664}\;-\;22.24A_{648}$$

(4) $$C_{\mathrm{carotenoids}}\;(µg/\mathrm{mL})=\frac{1000*A_{470}\;-\;2.13*Ca\;-\;97.64*Cb}{209}$$

### 2.5. Microbiological Tests

#### 2.5.1. Tested Microorganisms

All microorganisms were obtained from the culture collection of the Department of Microbiology, University of Food Technologies, Plovdiv, Bulgaria.

Pathogenic bacteria: *Staphylococcus aureus* ATCC 25923, *Listeria monocytogenes* NBIMCC 8632, *Klebsiella* sp. (clinical isolate), *Enterococcus faecalis* ATCC 29212, *Escherichia coli* ATCC 8739, *Salmonella enteritidis* ATCC 13076, *Proteus vulgaris* ATCC 6380, and *Pseudomonas aeruginosa* ATCC 9027. These strains were cultured on Luria–Bertani agar supplemented with glucose (LBG agar) at 37 °C for 24 h.

Spore-forming bacteria: *Bacillus cereus* ATCC 14579 and *Bacillus subtilis* ATCC 6633. Cultivation was performed on LBG agar at 30 °C for 24 h.

Yeasts: *Candida albicans* NBIMCC 74 and *Saccharomyces cerevisiae* ATCC 9763. *C. albicans* was cultured on LBG agar at 37 °C for 24 h, while *S. cerevisiae* was grown on malt extract agar (MEA) at 30 °C for 24 h.

Filamentous fungi: *Aspergillus niger* ATCC 1015, *Aspergillus flavus*, *Penicillium chrysogenum*, *Fusarium moniliforme* ATCC 38932, and *Mucor* sp. These fungi were cultivated on MEA at 30 °C for 7 days or until sporulation.

#### 2.5.2. Culture Media Preparation

Luria–Bertani agar medium supplemented with glucose (LBG agar).

Composition (g/L): tryptone—10.0; yeast extract—5.0; NaCl—10.0; glucose—10.0; and agar—15.0. pH7.5 ± 0.2. Sterilization: 121 °C/20 min.

Malt extract agar (MEA)

Composition (g/L): malt extract—30.0; mycological peptone—5.0; agar—15.0. pH 5.4 ± 0.2. Sterilization: 115 °C/10 min.

#### 2.5.3. Antimicrobial Testing

The antimicrobial activity of the extracts was determined by the agar-diffusion well method [35,36]. A total of 18 mL of pre-melted LBGH-agar medium, cooled to 40–45 °C and infected with the specified test microorganism (1.0 × 10^6^ cfu/mL for spores of mold fungi and 1.0 × 10^8^ cfu/mL for viable cells of bacteria and yeast), is poured into Petri dishes (*d* = 9 cm) and then placed on a level surface. The Petri dishes were left for one hour to solidify the agar. Using a cylindrical well puncher, 6 wells (*d* = 6 mm) were cut in the agar. A total of 60 μL of the tested solutions was instilled in triplicate. The Petri dishes are thermostated at temperature conditions corresponding to each test microorganism species for 24/48 h. The presence and degree of antimicrobial activity were determined by measuring the diameter of the inhibition zones around the agar wells. High antimicrobial activity is reported for inhibition zones of 18 mm or more; moderate activity is reported for inhibition zones between 12 and 18 mm; low activity corresponds to inhibition zone diameters of less than 12 mm.

#### 2.5.4. Minimal Inhibitory Concentration Determination

The minimal inhibitory concentration (MIC) is determined by the method of two-fold serial dilutions, according to Tumbarski et al. [37]. Two-fold dilutions of the tested solutions are prepared. The tested microorganisms are pre-inoculated into agar nutrient media in Petri dishes, and after the agar has solidified, six wells are drilled. Samples from each dilution are dropped in an amount of 60 μL into the wells, after which the Petri dishes are incubated under reduced conditions, depending on the type of test microorganism. The MIC value is determined as the lowest concentration of the extract which completely suppressed the growth of each test microorganism around the agar well.

### 2.6. Spasmolytic Activity Assesment

#### 2.6.1. Ex Vivo Experiments Involving Gastric Smooth Muscle Specimens

Male Wistar rats aged 3–4 months were supplied by the animal facility of the Medical University of Plovdiv, Plovdiv, Bulgaria. Animals were housed under conventional laboratory conditions (22 ± 2 °C; 12 h light/dark cycle) with free access to standard chow and water. Humane euthanasia was carried out by intraperitoneal administration of a supralethal dose of xylazine (2%, 10 mg/kg; Sigma-Aldrich, Germany) combined with ketamine (5%, 100 mg/kg; Sigma-Aldrich, Germany), in accordance with internationally recognized guidelines for the care and use of laboratory rodents [38,39].

All experimental procedures complied with the requirements of European Directive 2010/63/EU for the protection of animals used for scientific purposes and were approved by the Institutional Animal Ethics Committee. The study also adhered to relevant Bulgarian legislation, including the Animal Protection Act (SG No. 13/2008; amended SG No. 65/2020) and Ordinance No. 20/01.11.2012, which outlines the minimum standards for animal welfare and ethical conduct in experimental research, issued by the Bulgarian Ministry of Agriculture, Food, and Forestry.

#### 2.6.2. Evaluation of Spontaneous Contractile Activity in Ex Vivo Functional Assay on Rat Gastric Smooth Muscle Strips

Following induction of deep anesthesia, the stomach was removed via midline laparotomy. Circular strips of gastric smooth muscle (approximately 11.0–12.5 mm × 1.1–1.2 mm) were carefully dissected and prepared for physiological recording. Each strip was mounted in a 15 mL organ bath containing Krebs solution maintained at 37 °C and continuously aerated with a 95% O_2_/5% CO_2_ gas mixture [40]. One end of the tissue was secured to a fixed hook, while the other was connected to an isometric force transducer integrated into a Radnoti 8-Unit Tissue Organ Bath System (Model 159920, Radnoti, Dublin, Ireland). The tissues were allowed to equilibrate for 60 min, with the bathing solution renewed every 15 min. Contractile activity was continuously recorded using a PowerLab data acquisition system (ADInstruments, Dunedin, New Zealand) in combination with LabChart software and the Dose Response module [41,42,43]. During the adaptation period, tissue viability was evaluated twice by inducing contractile responses with acetylcholine (ACh, 10^−6^ M; Sigma-Aldrich, Germany). Spasmogenic effects of crude *Chlorella vulgaris* extracts, biogenic AgNPs synthesized using this extract, and AgNPs alone (controls) were assessed by cumulative addition. Contractile force was expressed in milliNewtons (mN) [44].

Throughout the study, we recorded several endpoints—contractile amplitude (mN), contraction frequency (cpm = contractions per minute), tonic shift relative to baseline (spontaneous) activity (mN), maximal effect (E_max_) obtained from cumulative dose–response curves, and the determination of submaximal effective concentrations (EC_submax_)—for each treatment. These parameters served as the basis for mechanistic interpretations and comparative analyses of the spasmogenic and spasmolytic properties of the tested preparations [45]. The number of smooth muscle strips used is indicated by n.

### 2.7. Antioxidant Activity Assesment

#### 2.7.1. DPPH Radical Scavenging Assay

The reaction mixture containing 2.85 mL of DPPH reagent (2,2-diphenyl-1-picrylhydrazyl) (Sigma-Aldrich, Merck, Munich, Germany) and 0.15 mL of the tested extract was kept at 37 °C for 15 min. The absorbance was measured at 517 nm against a blank (methanol). The antioxidant activity was expressed as mM Trolox equivalents (TE)/g of sample weight [46].

#### 2.7.2. ABTS Assay

The ABTS reagent is prepared according to the method described by Ivanov et al. [46]. The ABTS radical is generated by mixing aliquots of 7.0 mM 2,2′-azino-bis(3)-ethylbenzothiazoline-6-sulfonic acid (ABTS) in distilled water and 2.45 mM potassium persulfate in distilled water. The reaction is carried out for 16 h at room temperature in the dark, and the generated ABTS radical is stable for several days. Before analysis, 2 mL of the ABTS+ radical solution is diluted with methanol in a ratio of 1:30 (*v*/*v*) so that the final absorption of the working solution is within the range of 1.0 ÷ 1.1 at λ = 734 nm. For the analysis, 2.85 mL of ABTS+ reagent is mixed with 0.15 mL of the 95% tested solution. After keeping the reaction mixture at 37 °C in the dark for 15 min, the absorbance at λ = 734 nm against methanol is measured. Antioxidant activity is expressed as mM Trolox equivalents (TE) per g weight using a standard curve [46].

### 2.8. Statistics

The results were expressed as mean values and their standard deviations. All analyses were conducted in triplicate (n = 3), and a one-way ANOVA was performed to establish the significant differences in the results (Tukey, *p* < 0.05) using software SPSS Statistics 19.0 (SPSS Inc., Chicago, IL, USA).

## 3. Results and Discussion

### 3.1. Characterization

To comprehensively characterize the physicochemical properties of the synthesized AgNPs, a combination of spectroscopic, microscopic, and scattering techniques was employed. Particle size, morphology, crystallinity, surface chemistry, and colloidal stability were evaluated using FTIR-ATR, BF-TEM, XRD, DLS, and zeta potential measurements.

#### 3.1.1. FTIR-ATR Analysis

The biosynthesized AgNPs obtained using *Chlorella vulgaris* extract (*Chlorella*-AgNPs) in the present study exhibit predominantly spherical morphology and good colloidal stability, indicating effective surface passivation by microalgal biomolecules. These results are similar to those reported for AgNPs synthesized using *Spirulina platensis* extracts. In contrast, the broader particle size range observed for *Chlorella*-AgNPs may reflect differences in pigment composition and lipid-associated stabilizing agents specific to *Chlorella vulgaris*.

**Figure 1:**
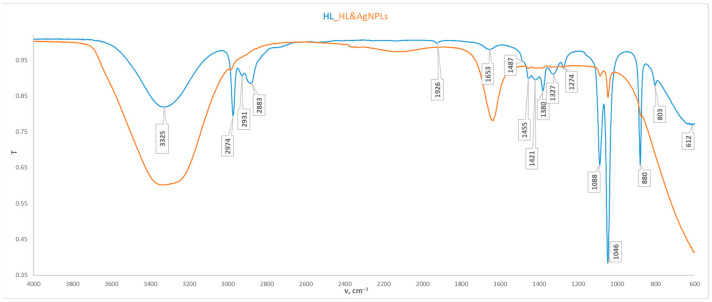
ATR spectra of *Chlorella vulgaris* (blue) and *Chlorella*-AgNPs (orange).

Figure 1 shows the overlapping ATR spectra of pure *Chlorella* extract, shown in blue, and that of the *Chlorella*-AgNPs in orange. While some bands overlap at certain characteristic intervals, significant differences are observed, indicating the effectiveness of the sample preparation. When comparing both spectra, it is evident that the broad band in the range 3000–3600 cm^−1^ is preserved, increasing in intensity, which is most likely due to the presence of -OH groups, as a result of the previous sample preparation. The band at 1637 cm^−1^ is slightly shifted compared to the *Chlorella*’s spectrum, but with greater intensity. The two bands for C-O-C—1085 cm^−1^ and 1045 cm^−1^ are present, but with significantly weakened intensity.

Comparison of the *C. vulgaris* spectrum (Figure 1) with the standard chlorophyll spectrum [47] shows that in certain characteristic intervals, they contain completely overlapping bands. This indicates the successful extraction process for chlorophyll dye. The *Chlorella v.* spectrum includes vibrational bands of some functional groups, which are traced in the corresponding characteristic intervals. There is a weak and broad band at 3325 cm^−1^, which corresponds to the stretching vibration of –OH groups. Symmetric and asymmetric -CH_3_ stretching vibrations of alkyl alkanes are observed at 2974 cm^−1^, 2931 cm^−1^, and 2883 cm^−1^, which are enhanced by the presence of C-H group stretching vibrations at 1380 cm^−1^. Weak stretching vibrations of C=C and C=N groups, indicative of chlorophyll pigments, are observed at 1653 cm^−1^ [48,49], C-O-C stretching vibrations of ester are observed at 1088 cm^−1^, and sharp stretching vibrations of -C-O of primary alcohol are observed at 1046 cm^−1^. The band at 880 cm^−1^ correlates with the out-of-plane bending vibration in –CH fragments.

#### 3.1.2. TEM Micrographs

**Figure 2:**
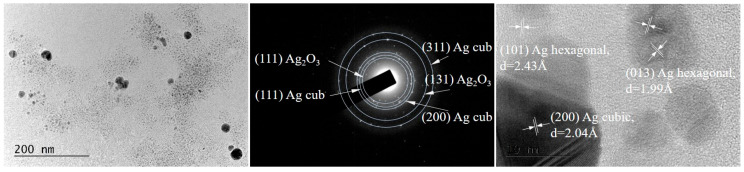
Bright-field TEM (BF-TEM) micrograph, as well as the corresponding SAED pattern and HRTEM image of *Chlorella*-AgNPs.

Figure 2 represents the BF-TEM micrograph at a magnification of 40,000× for biosynthesized AgNPs using *Chlorella* extract in 80% ethanol. The *Chlorella*-AgNPs show high electron density with predominantly spherical shape, suggesting isotropic growth, typical for Ag^0^ formed under mild reducing conditions from algal extracts. No clear faceted or crystalline edges are visible at low magnifications, but the darker contrast of the particles indicates a high degree of crystallinity. Most of these nanoparticles appear between 10 and 50 nm in diameter. Smaller nanoparticles of 5–10 nm and some larger aggregates (40–50 nm) are also observed. The estimated mean particle size is 40 nm. The apparent absence of extensive fusion between the particles confirms surface passivation by different phytochemicals in the extract. The presence of particles with larger diameters suggests partial agglomeration or incomplete control of nucleation that could be explained by the ethanol ratio. The higher ethanol concentration in the extract can denature protein caps, leading to incomplete stabilization and the occurrence of agglomeration during drying. The background shows a faint, continuous gray texture of organic residues, such as proteins, polysaccharides, or chlorophyll derivates, acting as capping and stabilizing agents in the process of AgNP formation.

The phase composition of the sample, determined by means of SAED, demonstrates the presence of two types of silver—cubic Ag, with lattice parameter a = 4.071 Å [COD Entry #96-150-9147], and hexagonal Ag, with a = 2.8862 Å and c = 10.0 Å [COD Entry #96-150-9195]—as well as a small amount of cubic silver oxide Ag_2_O_3_, a = 4.90400 Å [COD Entry #96-710-9248].

Lattice fringes are visualized in the HR-TEM images, providing further evidence for the crystalline state of the nanoparticles. The determined interplanar distances support the presence of two phases of Ag—cubic and hexagonal—identified by the electron diffraction.

#### 3.1.3. XRD

**Figure 3:**
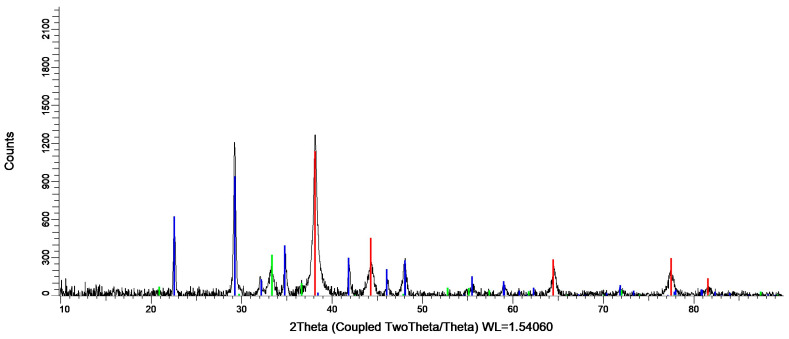
XRD analysis of the *Chlorella*-AgNPs. Red—cubic silver (PDF 00-004-0783), blue—silver nitrate (PDF 01-070-0198) and green—silver phosphate (PDF 01-075-5985).

The obtained *Chlorella*-AgNPs showed the distinguished 2θ peaks with the values of 38.20°, 44.35°, 64.50°, and 77.45° (Figure 3), corresponding to cubic silver (PDF 00-004-0783). The XRD analysis of the *Chlorella*-AgNPs is fully consistent with those obtained by Rajkumar et al. [28].

The crystalline character of the produced *Chlorella*-AgNPs has been demonstrated by the XRD data [50]. According to Jyoti and Singh (2016), the Miller indices (111), (200), (220), and (311) correspond to the four major peaks in the pattern, which are at 38.20°, 44.34°, 64.53°, and 77.40° [51,52].

#### 3.1.4. DLS and Zeta Potential

**Figure 4:**
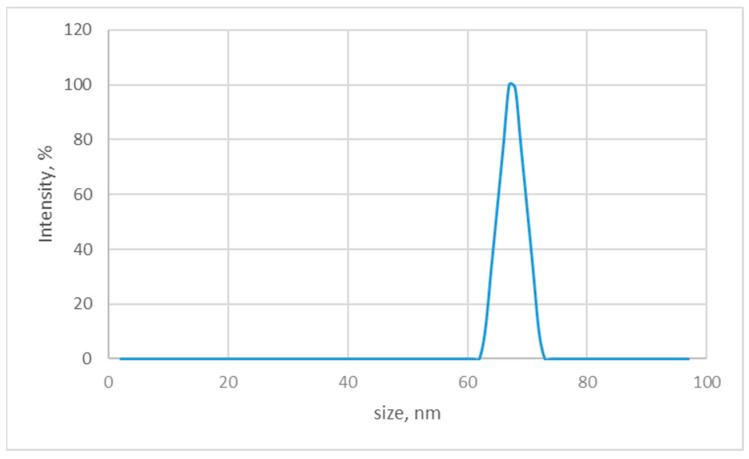
Dynamic light scattering histograms of *Chlorella*-AgNPs.

DLS verified the obtained *Chlorella*-AgNPs’ median size (Figure 4). Dynamic light scattering analysis revealed a monomodal size distribution with an average diameter between 60 and 80 nm and a PDI value of 0.206, indicating a relatively narrow size distribution. We noticed that the particle diameter exceeded that of the TEM measurement. To the best of our knowledge, in TEM, the particles are dried before measurement, whereas in DLS, the particle size is ascertained in a liquid solution. The increase in size in the DLS measurements may be attributed to the presence of organic matter shells that have the ability to absorb water. Furthermore, only the diameters of the particles without shells can be estimated when calculating the particle size based on TEM micrographs, which DLS is unable to discriminate. In TEM, the boundaries of *Chlorella*-AgNPs and shells are readily separated due to their differing contrast.

Flavonoids, tannins, saponins, phenolic acids, and other biomolecules on the AgNPs’ surface serve as capping agents due to their negative electric charge [53,54]. They usually have a negative charge and produce repulsive forces that keep AgNPs stable in solution and prevent their aggregation. Furthermore, the zeta potential of *Chlorella*-AgNPs has affected the distribution of particle sizes. *Chlorella*-AgNPs had a zeta potential of −24.9 mV. Our results are fully consistent with data reported by Michalec et al. [55], who obtained AgNPs with a zeta potential of −15.0 mV, indicating medium stability and a negative charge of particles. The results also correspond to those obtained by Noura El-Ahmady El-Naggar [56]. The authors found that the zeta potential value for AgNPs obtained from *Chlorella* extract was −31.3 mV. This value confirms the stability of the biosynthesized silver nanoparticles as reported by Hussein et al. [57].

### 3.2. Chemical Composition Changes

The glyceride oil content, total chlorophyll, and fatty acid composition of *Chlorella* extract and *Chlorella*-AgNPs were also examined. Table 1 represents the content of glyceride oil in the sample materials and the total chlorophylls in their isolated lipids.

#### 3.2.1. Fatty Acid Composition Changes

As presented in Table 1, after obtaining *Chlorella*-AgNPs, the content of glyceride oil rose significantly from 0.31% to 3.71%, representing a nearly 12-fold increase. A possible explanation for this increase could be the utilization of ultrasound-assisted extraction during the synthesis of the AgNPs. Ultrasonic cavitation disrupts the plant cell walls and membranes, facilitating the release of the oil. A similar tendency was observed in a previous study that examined the impact of nanoparticle formation on the matrices of *Spirulina platensis* [58]. Regarding the total chlorophylls, a significant decrease in their content was observed in the sample after the formation of silver nanoparticles, from 22.61 mg/kg to 10.74 mg/kg in the glyceride oil. A similar reduction in these components was reported by Tayemeh et al. [59], who found that the levels of these compounds decreased when exposed to increased concentrations of AgNPs and AgNO_3_ (1, 5, and 10 mgL^−1^).

The fatty acid composition of the examined samples is shown in Table 2.

Sixteen fatty acids were identified in the glyceride oils of both samples before and after the synthesis of the silver nanoparticles. The main fatty acids observed were palmitic acid, with contents of 32.3% and 30.2% in the lipids, respectively, followed by oleic acid (10.8 and 27.8%). The content of linoleic acid was significantly higher in the sample of *Chlorella*-AgNPs, amounting to 19.5% compared to 2.9% in the raw material. In both samples, stearic (8.0 and 5.0%), linolenic (6.1 and 5.2%), and palmitoleic (2.6 and 3.3%) acids were also identified in relatively high amounts. In the glyceride oil from the raw samples, the percentages of heptadecenoic (8.0%), 7,10-hexadecadienoic (7.5%), and margaric (5.8%) acids were also in larger quantities. The lipids isolated from the raw *Chlorella* species were characterized by higher amounts of capric (2.6%), undecanoic (2.6%), myristic (2.3%), myristoleic (2.0%), margaric (5.8%), 7,10,13- hexadecatrienoic (2.5%), heptadecenoic (8.0%), stearic (8.0%), α-linolenic (6.1%), and behenic (2.9%) acids than the oil from the material used for the production of the silver nanoparticles: 1.7%, 0.3%, 0.3%, 0.4%, 1.6%, 1.2%, 0.4%, 5.0%, 5.2%, and 0.3%, respectively. Interestingly enough, the content of saturated fatty acids decreased in the glyceride oil after the synthesis of the silver nanoparticles, with amounts dropping from 57.6% (in the raw material) to 40.6% (in the material after the production of AgNPs), which was in favor of the slight increase in the content of the mono- (from 23.4 to 31.9%) and polyunsaturated fatty acids (from 19.0 to 27.5%).

Kim and Hur [60] also reported that palmitic acid was the major component in the glyceride oil from *Chlorella vulgaris* cultured in autotrophic conditions at 26 °C, ranging from 18.1 to 23.5 μg/mg dry matter. The other main components were found to be α-linolenic (from 10.6 to 22.1 μg/mg) and linoleic acid (from 11.8 to 15.8 μg/mg). On the other hand, Jahromi et al. [61] established again that palmitic acid predominated in the lipid fraction (23.16%), but the amounts of linoleic and oleic acids were almost similar (17.93 and 17.80%, respectively). The same authors also reported levels of α-linolenic acid that were twice as high, reaching up to 12.42%. Tayemeh et al. [59] investigated the changes in the fatty acid composition of *Chlorella vulgaris* upon exposure to varying concentrations of ionic silver and silver nanoparticles. They reported that AgNPs had altered their composition, in which a significant decrease was observed in the content of myristoleic acid (C14:1-n5) and nervonic acid (C24:1-n9), while for the other fatty acids, no regular tendency in their concentration was noticed depending on the material’s response to exposure to silver ions and NPs.

The observed decrease in chlorophyll content and changes in lipid and fatty acid profiles following *Chlorella*-AgNP synthesis may arise from oxidative degradation, selective adsorption of biomolecules onto nanoparticle surfaces, or partial disruption of algal cellular structures during the synthesis. At present, these observations should be interpreted as correlative rather than mechanistic.

#### 3.2.2. Changes in Pigment Component

Prior to and during the synthesis of *Chlorella*-AgNPs, the amount of total pigment components in a 95% ethanol extract of *Chlorella* was determined. Chlorophyll a (Ca), chlorophyll b (Cb), and total chlorophyll (Ca+b), along with carotenoids, present significantly in the 95% ethanol extract of *Chlorella*, according to the data obtained (Table 3). Following the synthesis of AgNPs from the ethanol extract, a notable reduction in the quantity of chlorophyll a and chlorophyll b was noted because of their likely involvement in the redox process.

The optical characteristics of AgNPs can account for the observed rise in the measured amount of carotenoids following their production. The surface plasmon resonance (SPR) of silver nanoparticles is located between 400 and 450 nm in the UV–Vis spectrum. Depending on the size and shape of the particles, this SPR shifts and likely overlaps with the spectral region where carotenoids’ absorption maxima occur (450–500 nm), which has an impact on carotenoids’ spectral measurements [62].

### 3.3. Antimicrobial Activity

AgNPs have been synthesized utilizing *Chlorella vulgaris* as a reducing and stabilizing agent, in line with recent developments in green nanotechnology [55,63]. Due to their capacity to damage microbial membranes and produce reactive oxygen species, these biosynthesized *Chlorella*-AgNPs have strong antibacterial activity, frequently outperforming that of traditional antibiotics [64,65]. Additionally, compared to chemical approaches, the eco-friendly synthesis methodology decreases cytotoxicity and environmental impact [66,67].

In this context, the antimicrobial activity of *Chlorella*-AgNPs was evaluated by the agar-diffusion method (Table 4). The crude *Chlorella* extract and methanol used as a solvent for the samples did not show any inhibitory effect against all pathogenic and saprophytic microorganisms tested. The results were compared to AgNPs of equivalent silver content. We found that AgNPs have moderate activity only against *A. niger* with an inhibition zone of 14 mm.

The analyzed *Chlorella*-AgNPs showed a moderately pronounced inhibitory effect against all tested pathogenic microorganisms (Gram-positive—*S. aureus*, *L. monocytogenes*, *E. faecalis*; Gram-negative bacteria—*K. pneumoniae*, *E. coli*, *S. enteritidis*, *P. vulgaris*, *P. Aeruginosa*, and the fungus *C. albicans*), as well as against the spore-forming *Bacillus subtilis* and the yeast *Saccharomyces cerevisiae* with an inhibition zone of 12÷14 mm.

**Figure 5:**
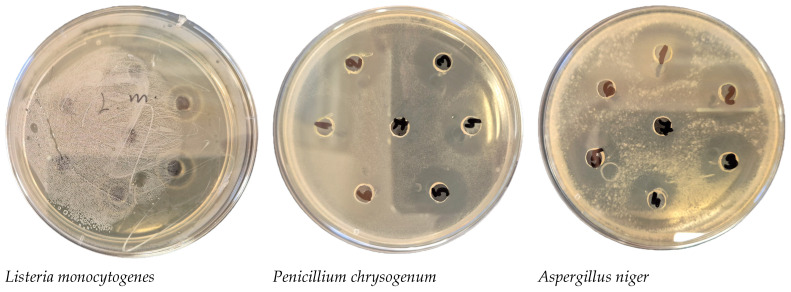
Selected Petri dish photos of antimicrobial and antifungal activity assay of *Chlorella*-AgNPs.

*Penicillium chrysogenum* and *Aspergillus niger* are more sensitive to *Chlorella*-AgNPs, with inhibition zones of 22 mm and 23 mm being established, respectively (Figure 5).

Using the minimal inhibitory concentration (MIC) assay, the antibacterial activity of *Chlorella*-AgNPs was determined against a panel of pathogenic Gram-positive and Gram-negative bacteria, yeasts, and fungi. The obtained MIC results demonstrate the broad-spectrum antibacterial activity of the *Chlorella*-AgNPs (Table 5).

The majority of the tested bacterial strains, including *Staphylococcus aureus*, *Listeria monocytogenes*, *Enterococcus faecalis*, *Escherichia coli*, *Salmonella enteritidis*, *Proteus vulgaris*, *Pseudomonas aeruginosa*, and *Bacillus subtilis*, showed high sensitivity to the *Chlorella*-AgNPs, with MIC values of 98.44 μg/mL. Due to its protective outer membrane structure, *Klebsiella pneumoniae* showed a significantly higher MIC value (196.87 μg/mL), indicating a considerably higher resistance. For *Saccharomyces cerevisiae*, a higher dosage (393.75 μg/mL) was necessary to suppress growth, while *Candida albicans* showed intermediate susceptibility with an MIC of 196.87 μg/mL. MIC values for pathogenic fungi, including *Aspergillus niger*, *Aspergillus flavus*, and *Penicillium chrysogenum*, ranged from 98.44 to 196.87 μg/mL.

The results indicated that *Chlorella*-AgNPs showed considerable antibacterial activity against the majority of examined bacterial strains (MIC = 98.44 μg/mL), while fungal strains showed higher MIC values, indicating reduced susceptibility. Strongest resistance was observed for *Mucor* spp.

Our results correlate with those obtained by Mohammad Soleimani and Maziar Habibi-Pirkoohi [63], who found that AgNPs from *Chlorella vulgaris* inhibited the growth of *S. aureus* at a concentration of 50 μg/mL. Michalec et al. [55] investigated the effect of AgNPs from *Chlorella* on chicken embryos and found that AgNPs inhibited *S. enterica* growth at concentrations higher than 6.75 mg/L.

The most common spoilage fungi are *Candida* spp., *Penicillium* spp., *Fusarium* spp., and *Aspergillus* spp. They have the ability to produce mycotoxins, which are extremely harmful to both humans and animals. Additional indicators of spoiling fungi include reduced germination, nutritional and chemical changes, and grain discolouration [68,69]. *Aspergillus* sp. is the most prevalent, accounting for up to 22% of air spore samples [70]. Only a small number of *Aspergillus* species are harmful to humans [71]. *Aspergillus* infections mostly affect the respiratory system.

In the presence of high humidity, oxygen, and carbon dioxide, spergillus spores can germinate in the lungs [72,73,74]. The most frequent species that causes aspergillosis is *Aspergillus fumigatus*, followed by *A. flavus* and *A. niger*. One of the most frequent infections that cause mycosis in humans is *A. niger*. Systemic invasive infections brought on by these molds can kill at least 50% of affected people [75,76]. A variety of nuts, fruits, vegetables, and grains are contaminated by *A. flavus*, which produces mutagenic and carcinogenic aflatoxins [77]. The recent increase in drug-resistant isolates of *Aspergillus* spp. has been associated with long-term exposure to antifungals [78].

In our results, the most pronounced effect of AgNPs was against *A. niger* and *P. chrysogenum*, with inhibition zones above 18 mm (respectively 23 mm and 22 mm). No activity was detected against *Mucor* spp. and *A. flavus*.

Overall, the antimicrobial activity of AgNPs obtained from *Chlorella* presents a viable approach to sustainable and natural antimicrobial treatments, particularly in light of the growing resistance to antibiotics.

### 3.4. Antioxidant Capacity

The antioxidant activity of the extract before and after the preparation of AgNPs was evaluated by two methods based on mixed hydrogen atom transfer and single-electron transfer mechanisms—DPPH and ABTS. The results obtained from the DPPH and ABTS methods showed that the 95% ethanol *Chlorella* extract possessed the highest antioxidant activity, while the 95% ethanol *Chlorella* extract + AgNPs exhibited lower values (Table 6). The decrease in antioxidant activity in the extract with silver nanoparticles can most likely be explained by the participation of biologically active components in the synthesis of AgNPs and, accordingly, leads to a decrease in their quantity [79].

The most abundant antioxidants in the *Chlorella* extract are carotenoids, chlorophyll *a* and *b*, phenophytes *a*, and lutein [80]. The decrease in the antioxidant capacity can be explained by the participation of these compounds in the AgNPs’ synthesis.

### 3.5. Spasmolytic Activity of Crude Chlorella vulgaris Extract vs. Biogenic AgNPs from Chlorella Extract

The present investigation aims to systematically compare the pharmacodynamic effects of three different treatments on isolated rat gastric smooth muscle: with crude *Chlorella vulgaris* extract, with *Chlorella*-AgNPs, and with AgNPs alone. The design is based on the hypothesis that biogenic nanoparticle formation may significantly modulate the bioactivity of the algal extract, potentially altering its spasmogenic or spasmolytic profile. The AgNPs alone serve as a control to distinguish intrinsic nanoparticle effects from those associated with the algal extract.

Spasmolytic activity refers to a reduction in contraction amplitude, frequency, or tonic tension, whereas spasmogenic effects denote an enhancement of contractile responses.

Microalga *Chlorella vulgaris* is well recognized for its rich composition of bioactive metabolites, including fatty acids [9], sterols [81], and antioxidants [82], which have been implicated in systemic anti-inflammatory and immunomodulatory effects [83].

Our previous studies have also begun to clarify the direct effects of *Chlorella vulgaris* on gastrointestinal smooth muscle physiology. Specifically, we [9] established that both the chemical profile (metabolite composition) and the physical characteristics (particle size) of *C. vulgaris* powders are critical determinants of their impact on gastric smooth muscle contractility ex vivo. Our findings showed that extracts from different sources induced tonic contractions via muscarinic acetylcholine receptors and L-type calcium channels, with the magnitude of these responses modulated by particle size and metabolite profile.

In addition, in vitro digestion and fermentation models have highlighted the capacity of *C. vulgaris* to modulate gut microbiota and increase short-chain fatty acid production, such as acetate, propionate, and butyrate—molecules that are known to influence gastrointestinal motility and smooth muscle behavior [84,85]. These findings support the hypothesis that *C. vulgaris* may exert direct effects on smooth muscle, possibly mediated through immune-neural–muscular crosstalk or by metabolic by-products.

Given the emerging interest in biogenic nanoparticle systems, synthesizing AgNPs using *C. vulgaris* extract may offer several advantages, including improved stability, altered surface chemistry, and enhanced biological interactions relative to the crude extract. *Chlorella*-AgNPs may also promote cellular uptake and modulate the release or accessibility of extract-derived metabolites [86,87]. While plant-mediated studies on AgNPs have shown promising in vivo efficacy [88,89], there is a notable gap in research specifically addressing how such biogenic nanoparticles influence smooth muscle contractility in an isolated organ bath setting.

Existing evidence indicates that AgNPs can modulate smooth muscle contractility in various organs, including the intestine, stomach, vasculature, and trachea. In intestinal models, these effects involve nitric oxide (NO) and serotonin (5-HT) signaling pathways, as demonstrated by Chávez Hernández et al. [90] in their ex vivo evaluation of PVP-coated AgNP in the rat small intestine. The authors also observed changes in contractility attributed to structural tissue alterations resulting from Ag accumulation in histological sections of the ileum. Similarly, Morsi et al. [91] reported that following the removal of AgNP and the addition of ACh, muscle strips regained contractile responsiveness, indicating that the effect is not destructive but rather regulatory and reversible—an important consideration for potential therapeutic applications. In our recent study, we further demonstrated that functionalized AgNPs affect the contractile activity of gastric smooth muscle, confirming that AgNPs can modulate smooth muscle contractions [92]. When AgNPs are used together with the anticoagulant phenindion, the drug’s relaxant effect increases significantly [93].

**Figure 6:**
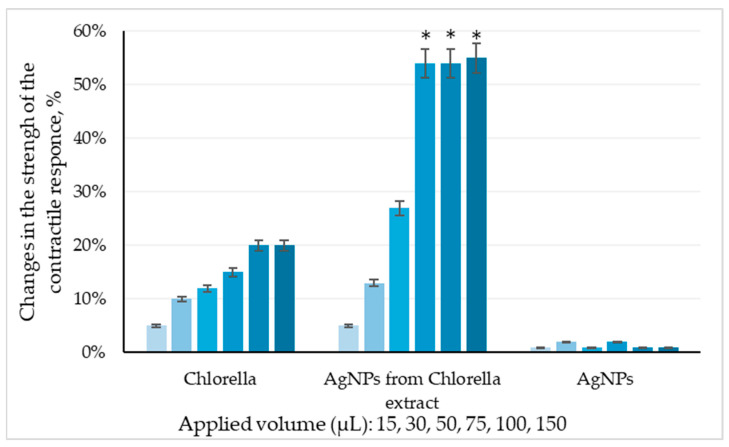
Percentage change in gastric smooth muscle contractile response strength to increasing volumes of crude *Chlorella* extract, AgNPs from *Chlorella* extract, and AgNPs (n = 8). Data are expressed as mean ± SD. Statistical analysis was performed using Student’s *t*-test. * *p* < 0.05 indicates statistical significance vs. crude *Chlorella* extract.

Our current idea is that *Chlorella*-AgNPs will have a significantly enhanced effect compared to the pure algal extract. This is particularly important for natural extracts, as they generally exhibit weaker effects compared to conventional drugs. We further hypothesized that *Chlorella*-AgNPs would exert a distinct spasmogenic or spasmolytic profile of the extract through changes in surface chemistry, metabolite presentation, or nanoparticle–tissue interactions. AgNPs alone, used as negative controls, did not significantly affect gastric smooth muscle contractile responses, confirming that observed effects are primarily attributable to the *Chlorella* component associated with biogenic nanoparticle formation (Figure 6).

Under the experimental conditions, both crude *Chlorella* extract and *Clorella*-AgNPs elicited a spasmogenic response, demonstrated as an increase in contraction amplitude and tonic tension relative to baseline spontaneous activity. No relaxant (spasmolytic) responses were observed for any of the tested treatments. Quantification was performed on amplitude (mN), frequency (cpm), and tonic shift parameters obtained from continuous recordings. The spasmogenic effect was volume-dependent, with *Chlorella*-AgNPs inducing significantly greater increases than the crude algae extract. In contrast, the individual application of AgNPs failed to modify spontaneous contractile activity, confirming the absence of intrinsic spasmolytic or spasmogenic effects. Positive control stimulation with ACh, 10^−6^ M resulted in a typical phasic–tonic contractile response, reaching a maximal strength of contraction of approximately 4.9 mN. This confirms the preservation of contractile capacity and tissue viability under the experimental conditions.

Quantitative analysis of contractile amplitude, frequency, and tonic shifts demonstrated that *Chlorella*-AgNPs not only enhanced the effect of *Chlorella* extract but also altered smooth muscle activity patterns, likely via modified receptor engagement or localized delivery.

Building on prior evidence that atropine and verapamil attenuate *C. vulgaris*-induced contractions through muscarinic acetylcholine receptors and L-type voltage-dependent calcium channels [9], we investigated receptor-mediated versus direct cytosolic calcium influx contributions. Results indicate that AgNPs from *Chlorella* extract modulate both qualitative and quantitative pharmacological profiles without confounding effects from the nanoparticles themselves. We were investigating this pathway following the previously used experimental protocol for the target compounds, and the results were confirmed [9].

Overall, *Chlorella*-AgNPs represent a promising approach to optimize its spasmogenic or spasmolytic activity in gastric tissue, with potential implications for mechanistic studies and therapeutic applications. Further research is necessary to clarify the underlying molecular mechanisms.

Considering potential translational applications, it is essential to recognize that AgNPs may exert long-term biological and environmental effects related to persistence, bioaccumulation, and release of reactive silver species. While the doses and conditions used herein are consistent with in vitro safety evaluations, extended exposure studies and environmental modeling will be required to fully assess ecological and toxicological risk profiles.

Finally, this study addresses a critical gap in the current literature. While *C. vulgaris* has been extensively investigated for its direct systemic and immunomodulatory effects, the impact of biogenic AgNPs synthesized using the extract on smooth muscle contractility remains unexplored. Understanding how pure *C. vulgaris* extract and its biogenic nanoparticle formulation influence gastrointestinal motility provides valuable insights for the potential applications of microalgae-derived nanoparticles in gastrointestinal pharmacology.

## 4. Conclusions

The current study shows that *C. vulgaris* extract can be effectively used as a reducing and stabilizing agent for the eco-friendly synthesis of AgNPs. Changes in lipid content, fatty acid composition, and pigment content during AgNP formation cause changes in the microalgal biochemical profile. The synthesized *Chlorella*-AgNPs demonstrate boosting antibacterial efficiency compared to the algal extract. The most pronounced effect was demonstrated against *A. niger* and *P. chrysogenum*. Based on the obtained results, we can conclude that *Chlorella*-AgNPs affect the contractility of the smooth stomach muscles ex vivo. The probable mechanism is due to changes in bioavailability, interactions with smooth muscle receptors, and signaling pathways.

This work advances the expanding field of green nanotechnology and encourages future research into microalga-based nanoparticles as novel options for nutraceuticals, functional food ingredients aimed at gastrointestinal problems.

Furthermore, the current findings highlight the potential of *Chlorella*-based AgNPs as modulators of smooth muscle activity. However, further in vivo safety, dose–response, and mechanistic studies are required before any translational, nutraceutical, or therapeutic applications can be considered.

## Figures and Tables

**Table 1:** Content of glyceride oil and total chlorophylls in the examined samples *.

Components	*Chlorella* Extract	*Chlorella*-AgNPs
Glyceride oil, % of the material	0.31 ± 0.02 ^a^	3.71 ± 0.08 ^b^
Total chlorophylls, mg/kg in the oil	22.61 ± 0.14 ^a^	10.74 ± 0.11 ^b^

* The results are expressed as mean values with their corresponding standard deviation (SD). Different small letters in the same row determine significant differences in the results (Tukey, *p* < 0.05).

**Table 2:** Fatty acid composition of the examined samples *.

Fatty Acids, %		*Chlorella* Extract	*Chlorella*-AgNPs
C _8:0_	Caprylic acid	1.1 ± 0.1 ^a^	1.2 ± 0.1 ^a^
C _10:0_	Capric acid	2.6 ± 0.1 ^a^	1.7 ± 0.2 ^b^
C _11:0_	Undecanoic acid	2.6 ± 0.1 ^a^	0.3 ± 0.0 ^b^
C _14:0_	Myristic acid	2.3 ± 0.0 ^a^	0.3 ± 0.0 ^b^
C _14:1_	Myristoleic acid	2.0 ± 0.1 ^a^	0.4 ± 0.0 ^b^
C _16:0_	Palmitic acid	32.3 ± 0.6 ^a^	30.2 ± 0.4 ^b^
C _16:1_	Palmitoleic acid	2.6 ± 0.1 ^a^	3.3 ± 0.1 ^b^
C _16:2_	7,10-Hexadecadienoic acid	7.5 ± 0.1 ^a^	1.6 ± 0.0 ^b^
C _17:0_	Margaric acid	5.8 ± 0.2 ^a^	1.6 ± 0.2 ^b^
C _16:3_	7,10,13-Hexadecatrienoic acid	2.5 ± 0.1 ^a^	1.2 ± 0.1 ^b^
C _17:1_	Heptadecenoic acid	8.0 ± 0.1 ^a^	0.4 ± 0.0 ^b^
C _18:0_	Stearic acid	8.0 ± 0.2 ^a^	5.0 ± 0.2 ^b^
C _18:1_	Oleic acid	10.8 ± 0.2 ^a^	27.8 ± 0.4 ^b^
C _18:2_	Linoleic acid	2.9 ± 0.1 ^a^	19.5 ± 0.2 ^b^
C _18:3_	α-Linolenic acid	6.1 ± 0.2 ^a^	5.2 ± 0.1 ^b^
C _22:0_	Behenic acid	2.9 ± 0.2 ^a^	0.3 ± 0.0 ^b^
Saturated fatty acids		57.6	40.6
Monounsaturated fatty acids		23.4	31.9
Polyunsaturated fatty acids		19.0	27.5

* The results are expressed as mean values with their corresponding standard deviation (SD). Different small letters in the same row determine significant differences in the results (Tukey, *p* < 0.05).

**Table 3:** Quantitive pigment content in *Chlorella* extract and *Chlorella*-AgNPs

	Ca [μg/g]	Cb [μg/g]	Ca+b [μg/g]	Carotenoids [μg/g]
*Chlorella* extract	4211.61 ± 5.07	856.68 ± 5.16	5068.30 ± 6.98	738.31 ± 6.15
*Chlorella*-AgNPs	2516.65 ± 7.43	608.33 ± 13.57	3124.99 ± 6.15	888.37 ± 5.66

**Table 4:** Antimicrobial activity of *Chlorella* extract and *Chlorella*-AgNPs.

	Inhibition Zone, mm	
Tested Microorganisms	*C. vulgaris* Extract	*Chlorella*-AgNPs
*Staphylococcus aureus* ATCC 25923	–	12 ± 0.0
*Listeria monocytogenes* ATCC 8632	–	14 ± 0.0
*Klebsiella pneumoniae* ATCC 13883	–	10 ± 0.0
*Enterococcus faecalis* ATCC 29212	–	12 ± 0.0
*Escherichia coli* ATCC 25922	–	12 ± 0.5
*Salmonella enteritidis* ATCC 13076	–	12 ± 0.0
*Proteus vulgaris* ATCC 6380	–	12 ± 0.0
*Pseudomonas aeruginosa* ATCC 9027	–	12 ± 0.0
*Candida albicans* NBIMCC 74	–	12 ± 0.0
*Bacillus subtilis* ATCC 6633	–	13 ± 1.0
*Saccharomyces cerevisiae* ATCC 9763	–	12 ± 0.0
*Aspergillus niger* ATCC 1015	–	23 ± 0.0
*Aspergillus flavus*	–	–
*Penicillium chrysogenum*	–	22 ± 0.0
*Mucor* spp.	–	–

**Table 5:** MIC of the biogenic *Chlorella*-AgNPs.

Tested Microorganisms	MIC, μg/mL
*Staphylococcus aureus* ATCC 25923	98.44
*Listeria monocytogenes* ATCC 8632	98.44
*Klebsiella pneumoniae* ATCC 13883	196.87
*Enterococcus faecalis* ATCC 29212	98.44
*Escherichia coli* ATCC 25922	98.44
*Salmonella enteritidis* ATCC 13076	98.44
*Proteus vulgaris* ATCC 6380	98.44
*Pseudomonas aeruginosa* ATCC 9027	98.44
*Candida albicans* NBIMCC 74	196.87
*Bacillus subtilis* ATCC 6633	98.44
*Saccharomyces cerevisiae* ATCC 9763	393.75
*Aspergillus niger* ATCC 1015	98.44
*Aspergillus flavus*	196.87
*Penicillium chrysogenum*	196.87
*Mucor* spp.	3150.00

**Table 6:** Antioxidant activity of AgNPs compared to the *Chlorella* extract.

	DPPH (mM TE/g)	ABTS (mM TE/g)
*Chlorella* extract	10.43 ± 0.18	1.01 ± 0.09
AgNPs	7.21 ± 0.35	0.31 ± 0.08

## Data Availability

Data are contained within the article.

## Abbreviations

The following abbreviations are used in this manuscript:

AgNPs	silver nanoparticles
TEM	transmission electron microscopy
DLS	dynamic light scattering
ATR	attenuated total reflection
GC	gas chromatography
FID	flame ionization detector
LBG	Luria–Bertani agar
*MEA*	malt extract agar
NO	nitric oxide
5-HT	serotonin
ACh	acetylcholine
XRD	X-ray diffraction
*Chlorella*-AgNPs	silver nanoparticles synthesized with *Chlorella vulgaris* ethanol extract
